# Report of Clinic of A. W. Calhoun, M. D.

**Published:** 1892-02

**Authors:** Jas. H. Latimer


### REPORT OF CLINIC OF A. W. CALHOUN, M. D.,

Professor of Diseases of Eye, Ear and Throat, Atlanta Medical College.

---

REPORTED BY JAS. H. LATIMER, JR.

---

I. G. E.: White, male, age twenty.

Diagnosis: Ulceration of cornea, deposit of albuminate of lead on cornea of both eyes.

### REMARKS.

Patient had probably had purulent ophthalmia and as a result ulcers of the cornea.

Doubtless the condition had been treated with acetate of lead, which, coming in contact with the albuminoid secretions of the eye, had formed an insoluble albuminate of lead, which was deposited on the cornea.

Dr. Calhoun said that he was glad of an opportunity of presenting a case of this character before the class, as it was one of the many instances in which a physician had injudiciously used acetate of lead in eye troubles, stating that while acetate of lead is one of our best astringents, it should never be used in eye troubles, especially where there is danger of ulcers, as invariably we would obtain just such results, which, besides ruining our patient's vision, would be a living monument of our unthinking treatment; and that while the ulcers may not be present at the time we make our diagnosis and prescribe, the possibilities are that they would creep out at any time, our patients awaking some morning with an abscess or an ulcer, and as a sequela these deposits, and as there are other astringents that will obtain for us the results wanted without this danger of a deposit, it is bad practice to use it.

The patient was given a solution of atropia (2 grs. to ounce) to allay pain and inflammation and cantharidal blisters applied to temples as counter-irritants, and instructed to return, when we will curette, and scrape off as much of the deposit as possible, and may be able to make pupil clear. If can't make pupil clear will make an iridectomy.

R. L. S.: White, male, age twenty-seven years, resides in Atlanta, Ga. Appeared before class Oct. 8th, 1891, claiming to have had secondary symptoms of syphilis before primary lesions appeared; was treated for syphilitic sore throat, even before chancre made its appearance. He claims it was three months after these lesions before the chancre appeared. The chancre covered the entire glans penis and a caustic was applied. About the same time acute articular rheumatism of ankles, also muscular rheumatism in left arm appeared. Patient gave no history of rheumatism in parents. Had been treated by several physicians for *Rheumatism*, but grew worse; suffering more in changeable weather, much worse at night than at day, not abating at all until antisyphilitic remedies were resorted to. Right hand in the meantime had become swollen to the extent that could not hold a pencil, and at time he presented himself had not subsided. Patient had at the time he presented himself a beautiful crop of typical mucous patches covering entire soft palate and sides of mouth, which was accompanied with nystagmus of both eyes, also leuchoma on left cornea. Patient was immediately placed on mercurial treatment. Proto-iodide of mercury was given, commencing with one-fifth grain doses three times daily. No treatment was directed for the other troubles.

Results: First week, mucous patches much better; dose was increased to two-fifths grain three times a day.

Second Week: Mucous patches better; increased dose by one-fourth grain three times daily.

Fifth Week: Continues to improve; ordered to continue same dose.

Sixth Week: About same as fifth, but three spiculæ of the hard palate had become necrosed and working through soft textures of hard palate into mouth, leaving openings through which a probe could be passed, touching the bone.

December 4th: Mucous patches were all gone, but the openings to palate bone were still open, but slightly healing.

December 17th: The openings were better, but they were still open; same treatment continued.

January 7th, 1892: About the same; treatment changed; was put on iodide of potash and mercury; saturated solution as follows:

R. Kalii iod., ℥ss.
Hydrarg. bichlo., gr. i.
Tr. gentian,
Aquæ aa. ℥i.
M. Ft. sol.

SIG.—10 increased to 75 drops three times a day, largely diluted after meals.

January 14: Openings in roof not closed but improved; had a hordeolum on lower palpebra—opened and evacuated.

REMARKS: The nystagmus is in all probability congenital; could get no history of the leuchoma; it possibly is not the result of any cause due to syphilis, but to some corneal troubles, independent of syphilis.

---

Dr. Wm. L. Russell has recently been appointed to the position of Attending Physician in the Department for Diseases of the Heart and Lungs at Demitt Dispensary, New York.

